# Application of Bayesian Regression for the Identification of a Catchment Area for Cancer Cases in Dogs and Cats

**DOI:** 10.3389/fvets.2022.937904

**Published:** 2022-07-25

**Authors:** José Manuel Díaz Cao, Michael S. Kent, Ruwini Rupasinghe, Beatriz Martínez-López

**Affiliations:** ^1^Center for Animal Disease Modeling and Surveillance (CADMS), Department of Medicine & Epidemiology, School of Veterinary Medicine, University of California, Davis, Davis, CA, United States; ^2^Center for Companion Animal Health and the Department of Surgical & Radiological Sciences, School of Veterinary Medicine, University of California, Davis, Davis, CA, United States

**Keywords:** canine, feline, cancer, catchment area, Bayesian models

## Abstract

Research on cancer in dogs and cats, among other diseases, finds an important source of information in registry data collected from hospitals. These sources have proved to be decisive in establishing incidences and identifying temporal patterns and risk factors. However, the attendance of patients is not random, so the correct delimitation of the hospital catchment area (CA) as well as the identification of the factors influencing its shape is relevant to prevent possible biases in posterior inferences. Despite this, there is a lack of data-driven approaches in veterinary epidemiology to establish CA. Therefore, our aim here was to apply a Bayesian method to estimate the CA of a hospital. We obtained cancer (*n* = 27,390) and visit (*n* = 232,014) registries of dogs and cats attending the Veterinary Medical Teaching Hospital of the University of California, Davis from 2000 to 2019 with 2,707 census tracts (CTs) of 40 neighboring counties. We ran hierarchical Bayesian models with different likelihood distributions to define CA for cancer cases and visits based on the exceedance probabilities for CT random effects, adjusting for species and period (2000–2004, 2005–2009, 2010–2014, and 2015–2019). The identified CAs of cancer cases and visits represented 75.4 and 83.1% of the records, respectively, including only 34.6 and 39.3% of the CT in the study area. The models detected variation by species (higher number of records in dogs) and period. We also found that distance to hospital and average household income were important predictors of the inclusion of a CT in the CA. Our results show that the application of this methodology is useful for obtaining data-driven CA and evaluating the factors that influence and predict data collection. Therefore, this could be useful to improve the accuracy of analysis and inferences based on registry data.

## Introduction

Data coming from laboratories or hospitals constitute an important source of information to assess incidence, relative risks, or to identify risk factors or temporal trends for several diseases. The usefulness of these data sources is especially evident in the epidemiological research of cancer in dogs and cats. In these species, cancer is a major pathology and constitutes the leading cause of death in dogs (1–3) as well as one of the main causes of mortality in cats (4, 5). Research based on the information collected from hospital cancer registries has substantially contributed to increased knowledge on the risk and incidence of different types of cancer in these species, and thus, on how to prevent and combat this pathology (6–9).

However, these data are not exempt from biases. Cases are not randomly obtained from the population but rather different factors, such as the distance to the center or the socioeconomic status of owners, among others, may influence first, the decision of looking for veterinary care and seek specialty care and the choice of a center instead of others in a competitive environment (10–12). Misidentification of the underlying population that is providing the data and the degree of underreporting affect the accuracy and reliability of any subsequent analysis (7, 13). A first step in addressing this issue is to determine the area from where the cases collected by a hospital are drawn, i.e., its catchment area (CA). Earlier and simpler attempts to estimate CAs in human medicine were based primarily on establishing a threshold distance (e.g., the spatial distance to the hospital or travel-time distance by road) and including in the CA those geographic units within this threshold (14–17). Another approach is including areas within a CA if a geographical unit contributes a threshold percentage of the center's total patients (18, 19).

The main problem with these approaches is the subjectivity and arbitrariness in the establishment of these thresholds for parameters such as the proximity of clients. These parameters are reported as highly variable in humans, depending on hospitals and hospital services (20–22). As a result, different statistical methodologies have been proposed as alternative data-driven approaches to estimate CAs for hospitals. Some of these consisted of the implementation of clustering methods based on K-means (20) or on local spatial scans, (e g., SaTScan) (23–25). Furthermore, more recently, generalized additive models (26) or Bayesian models (25, 27) have been successfully used to describe hospital service areas.

However, no similar statistically oriented approaches have been applied in veterinary medicine, even though they may provide similar benefits. A precise determination of the origin of cases and the factors that affect their reporting will improve the accuracy of subsequent inferences and analyses. This is of great interest in cancer in dogs and cats due to the significance of this pathology. The Bayesian analysis offers not only the possibility of estimating the CA in a probabilistic framework but also allows for making statistical inferences and considering the influence of covariates (25). Therefore, in this study, we aimed to establish the CA of a hospital for the cancer cases submissions in dogs and cats using Bayesian models. In addition, we also evaluated the influence of potential factors related to the constitution of the CA.

## Materials and Methods

### Study Area and Data Collection

We obtained records of visits of dogs and cats from the electronic medical record system of the Veterinary Medical Teaching Hospital at the University of California, Davis (Davis, California, United States) for the years 2000–2019. This database includes patient demographics and clinical data as well as the coordinates of the patients' domicile. As the registry included visits from distant areas, we made an initial subset of this database by selecting those records from a 145-mile (233 km) circular buffer zone around the hospital to rule out distant sporadic submissions in the whole period (20 years). This area was empirically established and assumed to represent a reasonable distance that an owner could drive to seek care and was used in previous studies (28). Patients from census tracts (CTs) within or intersecting the circle were included accounting for 2,707 CT from 40 counties. This subset represented 91.6% of the entire database and was used to obtain counts of two types of records: records with a diagnosis of cancer (cancer cases) and total records regardless of the diagnosis (visits). Cancer diagnosis was made by the overseeing clinician based on histology or cytology, similarly to other studies using veterinary medical databases (3, 29, 30). In order to avoid overestimating visits due to repeated visits of the same animal, only one record of the same animal per year was considered. If an animal was diagnosed with cancer in a calendar year, it was counted as one cancer record and additional records of this animal in that year were excluded (e.g., following-up visits, visits for other reasons, etc.). As a result, the dataset contained 232,014 visit registries (184,192 dogs and 47,822 cats) and 27,390 registries of diagnosed cancer cases (22,090 dogs and 5,300 cats).

### Statistical Analysis

Hierarchical Bayesian models were used to estimate the CA of cancer cases and visit records. Hierarchical modeling is written in levels that take account of the clustering of the population, such as CTs. A Bayesian approach in hierarchical methods presents further benefits in terms of flexibility of the models, for example, to capture possible correlations among the observations of interest, which can be addressed using conditional models (31), and which are not uncommon with administrative separations, when they do not actually prevent the movement across them (“edge effect”). These methods allow the calculation of exceedance probabilities which are useful when assessing the localized spatial behavior of the model and the detection of clustering (32).

The Bayesian regression models were fitted using Stan with the “brms” package (33) for the software R (34). Records were grouped into four periods (P1–4) to explore temporal variability: 2000–2004, 2005–2009, 2010–2014, and 2015–2019. Species and periods were included as fixed factors with CT as a random effect. The number of cases and visits observed were expected to follow a Poisson distribution with the expected number of cases/visits in each CT as the offset. The dog and cat population in CT were calculated from the estimate of the number of dogs and cats per household for California reported by the American Veterinary Medical Association (35) (average of 1.6 dogs and 1.7 cats per household). We multiplied these values by the number of households according to the United States Census (36) to estimate the population. As a result, the expected count for each observation was the total cases/visits count divided by the population in the CT and then multiplied by the population in the CT for each given combination of species and period.

Different alternative distributions were considered to fit the data by running models with different likelihoods: zero-inflated Poisson, negative binomial, zero-inflated negative binomial, Conway-Maxwell Poisson, and zero-inflated Conway-Maxwell Poisson. In addition, two different priors were considered for the random effects: an exchangeable prior and a conditional autoregressive (CAR) prior (31). The difference between these approaches is that the former assumes that random effects are independent, while CAR models take into consideration the correlation among neighbors, thus assuming that observations from neighbor CT tend to have similar values. The final selected model was a negative binomial with a CAR prior and was specified as:


$$\begin{array}{l} \;Y_{ij}=\{\begin{array}{ll} 0 & \;with\;probability\;\pi_{ij}\\ NB\left(\mu_{ij}\right) & \;with\;probability\;(1-\;\pi_{ij}) \end{array}\\ \;\mu_{ij}=\;e_{ij}\;\times \;R_{ij}\\ \log\left(\frac{\pi_{ij}}{1-\;\pi_{ij}}\right)=\;\beta_{0}+\;\beta_{1}\;Species_{ij}+\;\beta_{2}\;Period{\;}_{ij}+\;v_{ij}\\ \;\log\left(R_{ij}\right)=\;\beta_{0}+\;\beta_{1}\;Species_{ij}+\;\beta_{2}\;Period{\;}_{ij}+\;v_{ij} \end{array}$$

where *Y*_*ij*_ is the count of cases (cancer cases or visits) in CT *i* with covariate *j*, μ_*ij*_ is the unknown mean count, π_*ij*_ is the probability of diagnosis, *e*_*ij*_ is the expected count in CT_*i*_ covariate *j* over all CT, R_*ij*_ is the relative risk and ν_*ij*_ is the CT-specific random effect and β are the regression coefficients for each predictor. The intrinsic CAR distribution may be expressed as


$$\begin{array}{l} v_{i}|v_{\left(l\;\neq i\right)}\;\sim \;N\;\left(\left({\bar{v}}_{i}\right),\;1/\left(m_{i}\;\tau \right)\right) \end{array}$$

where *m*_*i*_ is the number of adjacent counties for CT *i*.

The models were run with four Markov chains with 4,000 iterations and a burn-in of 25%. Chain convergence was assessed using the packages “shinystan” (37) and “bayesplot” (38). Diagnostics were made using the potential scale reduction statistic (39), the ratio of the effective sample size to the total sample size drawn from the posterior distribution, and trace plots of Markov chain Monte Carlo. In summary, for each case/visit database, we run 12 candidate models. The selection of the final model was assessed by Bayesian leave-one-out cross-validation (40) using the package “loo” (41) and was compared based on the expected log pointwise predictive density (ELPD) using the Pareto smoothing importance sampling. This has been shown to be a robust method for model evaluation (42).

The methodology for estimating CA was similar to that previously described by Wang and Wheeler (25) using exceedance probabilities. This parameter was defined as the probability that the relative risk (RR) of each CT exceeded the null value (RR = 1) and was obtained from the posterior sample distribution. The threshold of the exceedance probability for a CT to be considered as part of the CA was set to 0.90 as this is a conventional value in disease mapping (32). This means that a CT exceeding a RR = 1 in 90% of the iterations was considered to be within the CA. We additionally explored other solutions using different thresholds for the exceedance probability including 0.95, 0.85, and 0.80.

We analyzed the influence of the average household income in the CT and the distance to the hospital as possible determinants for a CT to be included in the CA obtained in the final method. To do this, we run binary logistic regression models. The outcome of these models were belonging to the AC or not and we progressively included as predictors distance to the hospital, median family income, and an interaction term between the aforementioned factors. Average household income values per CT were obtained from the United States census, and distances were calculated as the driving distance from the centroid of the CT to the hospital using the package “gmapsdistance” (43). The values of these two predictors were centered on the mean for the analysis. A ROC analysis was subsequently performed to evaluate the accuracy of the logistic model with these factors to predict the resulting CA. The function “glm” and the package ROCR (44) were used in this part of the analysis. ANOVA tests were used to analyze mean differences between groups. The finally presented model was specified as:


$$\begin{array}{l} logit\;\left(p\right)=\;\beta_{0}+\;\beta_{1}\;Distance\;to\;the\;hospital\\ \;+\;\beta_{2}\;Average\;household\;income\\ \;+\;\beta_{3}\;Distance\;to\;the\;hospital\\ \;\times Average\;household\;income \end{array}$$

where *p* is the probability of being included in the CA.

## Results

**Figure 1:**
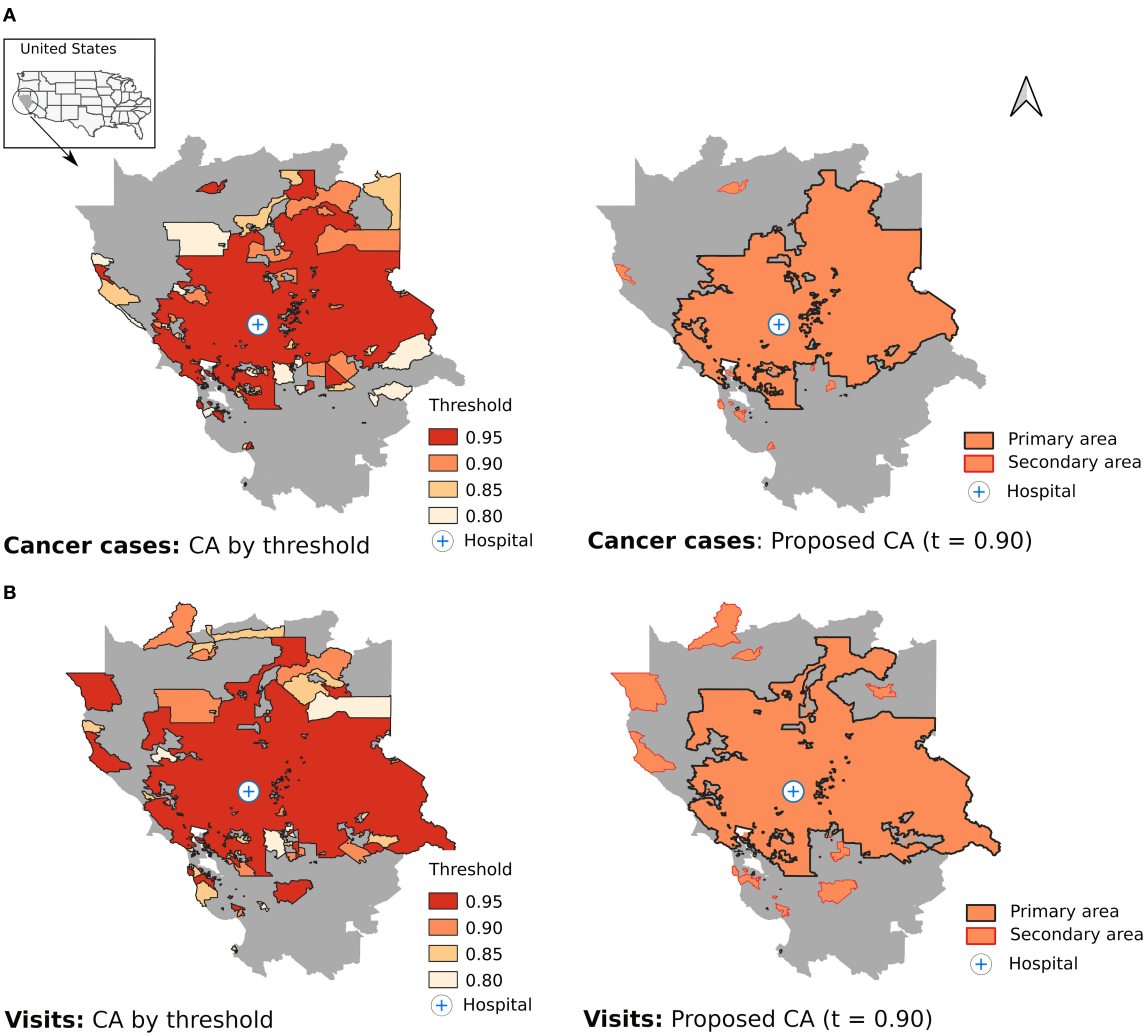
Estimation of the catchment area using different thresholds for the exceedance probabilities (left) and proposed catchment area with a threshold of 0.90 (right) for cancer cases **(A)** and visits **(B)**.

The models finally selected were those with a zero-inflated binomial distribution and a CAR prior for both cancer cases and visits. This model presented the best fit based on ELPD The proposed CAs were based on the solution obtained with a threshold for the exceedance probability of 0.90 (Figure 1). Thus, 936 CTs were included in the cancer cases CAs (34.6% of the CT in the buffer area) and 1,064 in the CA based on visit records (39.3%). These CAs accounted for 75.4% of the cancer cases and 83.1% of the visits registered by the hospital, while they represented only 34.6% and 39.3% of the total CT in the study area. The concordance in the classification obtained by both methods was very high (91.3%; 2,471/2,707), and the discrepancies were mainly due to the fact the CA obtained from visit records was broader: 182 CT included in the visits CA were not present in the cancer cases CAs (6.7 %). The opposite only occurred 54 times (2.0 %). Solutions with thresholds other than 0.90 are also shown in Figure 1, but no large variations were observed. Thus, the most liberal scenario (threshold of 0.80) only included 141 and 108 additional CTs for cancer cases and visits, respectively, and represented only a small increase in the CA compared to the solution at 0.90 (Figure 1).

As expected, the hospital was located in a central position and the CAs consisted mostly of a set of contiguous CTs around it (Figure 1, primary area) with a small proportion of CTs with no geographical connection with the primary area (8.2% for cancer cases and 8.6% for visits; secondary area). The models did not identify 301 and 245 CTs within the primary area boundary as members of the CAs for cancer cases and visits, respectively.

**Figure 2:**
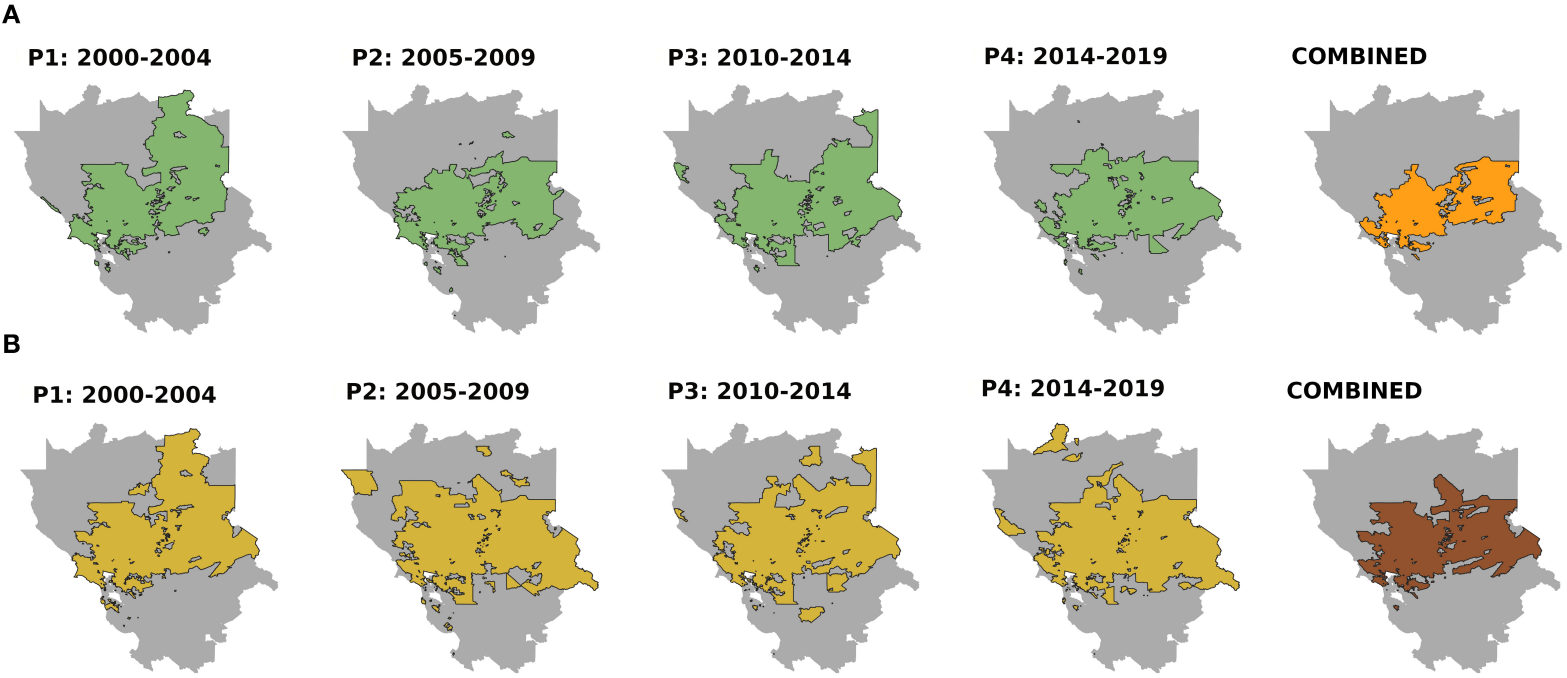
Catchment area for each period (P1–4) and combining only the census tracts included in the catchment areas of all periods (combined) for cancer cases **(A)** and visits **(B)**. Threshold of the exceedance probability = 0.90.

The parameters of the models are shown in Table 1. Dogs had a higher number of records of both cancer cases and visits (RR = 2.6 and 3.0, respectively, compared to cats). Regarding time period, an increase of records was observed in the study area during P3 in both models (RR = 1.2 and 1.1, respectively) compared to P1, but the trend observed in the models was different in P4. The estimation of the CA obtained in each period was very similar (Figure 2) showing concordances between periods that ranged from 85 to 91%.

**Table 1:** Results of the factors included in the Bayesian hierarchical models to determine the catchment area for cancer cases and visits.

		**Cancer cases**				**Visits**			
		**σ**^**2**^ R.E.*= **0.01**				**σ**^**2**^ **R.E**. **=** **0.01**			
**Variable**	**Category**	**B**	**SE**	**RR**	**CI 95**	**B**	**SE**	**RR**	**CI 95**
Species	Feline	**				**			
	Canine	0.9	0.02	2.6	2.4–2.6	1.1	0.01	3.0	2.9–3.1
Period	P1: 2000–2004	**				**			
	P2: 2005–2009	−0.04	0.02	0.9	0.9–1.0	0.02	0.01	1.0	1.0–1.0
	P3: 2010–2014	0.2	0.02	1.2	1.2–1.3	0.1	0.01	1.1	1.1–1.2
	P4: 2015–2019	0.01	0.02	1.0	1.0–1.1	0.3	0.01	1.3	1.3–1.3

*σ^2^ R.E.: variance of the random effect (census tract).

**Reference category.

*B, coefficient; SE, standard error; RR, relative risk; CI 95, Confidence interval at 95 %*.

**Figure 3:**
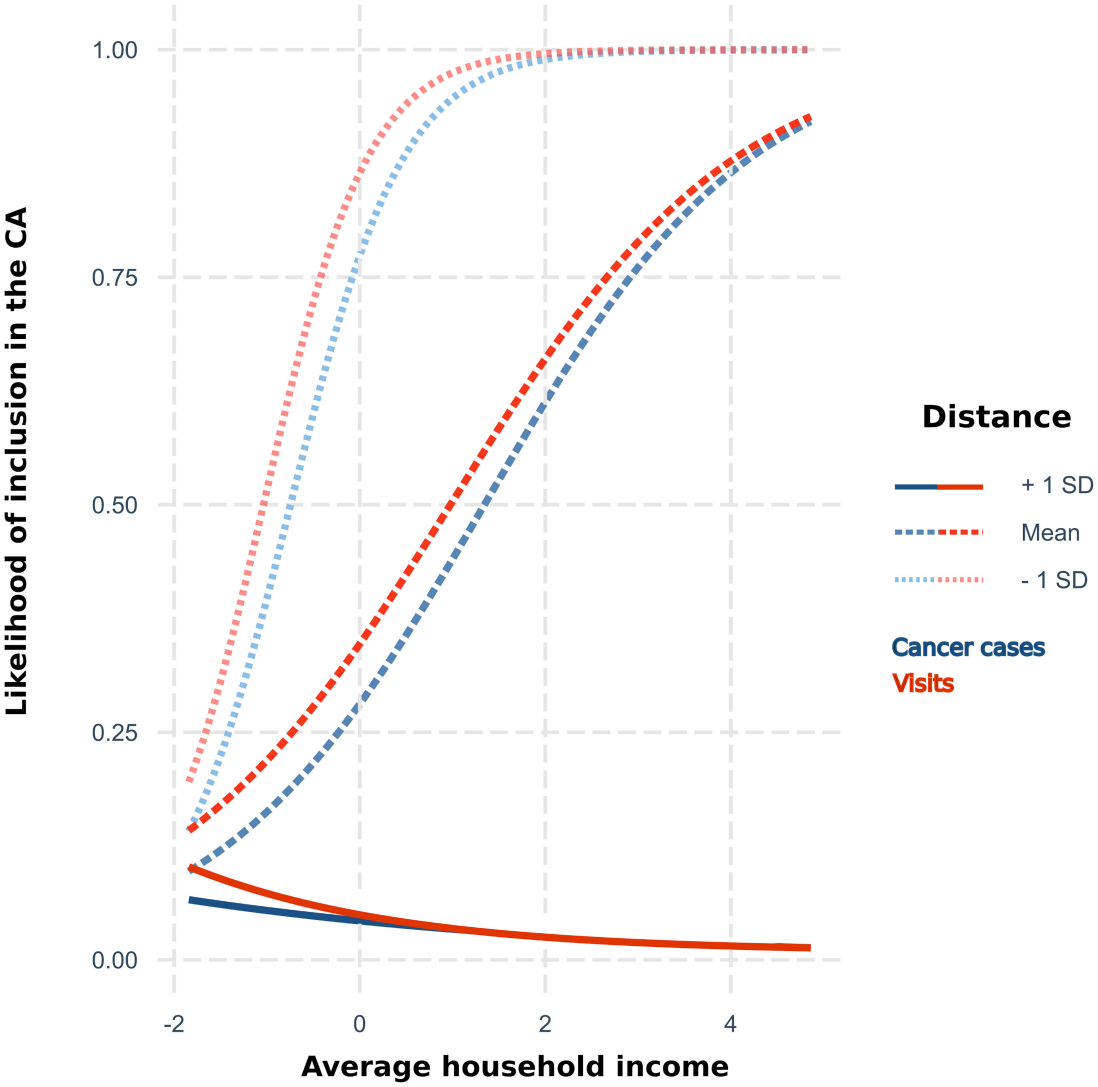
Likelihood of inclusion in the proposed catchment area by average household income [centered to the mean ($ 77,571)] for three levels of distance (centered, mean = 125.6 km) to the hospital according to the results of the logistic regression for cancer cases and visits.

The logistic regression identified both average household income and distance to the hospital as predictors of the CA (Table 2), and the model that included an interaction term presented the best fit. As expected, the probability that a CT would be included in the CA decreased with distance (OR = 0.1) and the CT with higher annual household income were more likely to be included (OR = 2.0). Distance modulated the effect of the income. Thus, the effect of the income decreased (OR = 0.4) with the distance to the hospital, being insignificant in CT located over long distances (Figure 3). The ROC analysis showed that models that only included distance had a high accuracy to predict the inclusion of CT in the CA: area under curve (AUC) = 0.83; but the accuracy increased to 0.87 including income and the interaction term (same values in both CAs). CTs within the boundaries of the primary area that were not estimated in the CAs by the models also showed a significantly (*p* < 0.05) lower average household income: $ 61,886 *vs*. $ 82,197 in cancer cases CAs and $ 58,901 *vs*. $ 79,840 in visits.

**Table 2:** Results of the logistic regression to analyze the associations between distance to hospital and average household income with the inclusion in the catchment area (CA) estimation for cancer cases and visits.

**Cancer cases**						
**Variable**	**Deviance**	**Estimate**	**OR**	**CI 95%**	**Mean in all the census tracts (SD)**	**Mean in CA *vs*. outside CA**
Distance to hospital (km)	978.2	−2.2	0.1	0.1–0.1	125.6 (57.4)	86.1 vs. 146.5
Avg income* (x 1,000 $)	48.4	0.7	2.0	1.8–2.3	77.6 (35.5)	84.1 *vs*. 74.1
Avg income: distance	135.8	−0.9	0.4	0.3–0.5		
Visits
Distance to hospital (km)	1042.4	−2.5	0.1	0.1–0.1	125.6 (57.4)	86.3 *vs*. 151.1
Avg income (x 1,000 $)	153.1	0.7	1.9	1.7–2.2	77.6 (35.5)	81.8 *vs*. 74.8
Avg income: distance	161.1	−1.1	0.3	0.2–0.4		

*Avg income, average household income.

*OR, odds ratio; CI 95 %, confidence interval at 95 %; SD, standard deviation*.

## Discussion

The identification of a hospital CA has been a historical challenge (45) that has not been well explored in veterinary medicine. In human medicine, CAs have been traditionally determined by including the local area surrounding the center (46) or capturing the geographic area from which clients come to the center (19). However, some of the criticism of traditional approaches is the arbitrariness of establishing preset distance/visits thresholds (20). Furthermore, setting the same thresholds for different situations is not realistic and they need to be revised according to different hospitals or hospital services (16). In fact, it is known that the CA can be affected by different factors (e.g., geographical, cultural, or historical) with variable effects, depending on the specialty or socioeconomic characteristics of the potential clients in the area (20, 47). For example, it is well known that catchments of urban or rural environments may be different and may also differ based on the type of disease (45, 48).

With this in mind, in this study, we have applied a data-driven methodology to identify the CA and address these issues by providing a framework that can be extrapolated to other centers, services, and conditions (25). The solution proposed here was successful in accomplishing those requirements of distance to the center and capturing the main areas that provide more clients to the center since we identified a geographical area close to the hospital that collects the vast majority of the visits. In order to explore the impact in the estimation of considering diverse outcomes, we additionally estimated the CA for visits. Despite the good agreement between both CAs, the solution for visits was slightly wider and included a higher number of CTs. The significantly lower average income in the CA estimated for visits than found for the CA of cancer cases (81,800 *vs*. 84,100; Table 2) could help explain these differences. Owners' income has been identified as an influential factor in the under-ascertainment of cancer in companion animals (10, 49). Pet owners with lower incomes may be less likely to seek a veterinary cancer diagnosis or to present their animal for regular veterinary check-ups, which could result in fewer cancer diagnoses (10, 11). In contrast, the number of reasons for visiting a hospital is varied and may include less expensive than seeking treatment for cancer, thereby having a less limiting effect on hospital presentation. The possibility of identifying suspicious areas of undercounting by comparing different types of CAs may be a secondary value to the methods used here and could be useful to propose measures and approaches to consider this issue in epidemiological studies.

The methodology we used also allows for assessing the influence of multiple variables on the composition of a CA. Dogs showed a higher risk of cancer cases and visits, which is consistent with previous research for both cancer cases (13, 50) and veterinary visits (35). Moreover, the latter may contribute to a further under-ascertainment of cancer cases in cats (49). The variation found by time period is not unexpected since CA is expected to vary over time (20). Thus, in addition to an average solution for the whole period of study (2000–2019) (Figure 1), we also provide estimations for each period as well as those CTs that were included in the CA in all the periods (Figure 2). The high concordance (85–91%) of these estimations suggests a high consistency of the results. Variation between periods may simply reflect changes in different socioeconomic factors (e.g., economic context, trends in social behaviors and companion animal ownership, change in the type of services offered by a hospital, prices, etc.).

However, this temporal variation mainly affected the CT not contiguous to the primary area. The inclusion of these CTs in the CA is not a surprising finding, since an area may be a major contributor to the cases registered by the hospital despite the lack of physical contiguity. For example, this may happen if some CTs present a higher socioeconomic status than their neighbors or if patients are frequently referred from local clinics for less available procedures (7, 8). Nevertheless, it cannot be ruled out that some of these CTs in the secondary area may be artifacts due to random variation. These CTs corresponded mainly to large and sparsely populated areas and these characteristics may favor the appearance of outliers (51) and lower statistical stability (52). Therefore, we preferred being conservative and assigned them to a secondary area because, in these circumstances, a random increase on the records might overestimate the relative contribution of those CTs to the hospital. In the opposite situation, we found that some CTs were not included in the CA despite being surrounded by the CA. The significant lower income found in these CTs may be again the explanation and indicate an under-ascertainment of cases from these areas due to economic reasons, resulting in a lower contribution in records to the hospital and then in the subsequent exclusion from the CA. This shows the potential of this workflow to identify areas with the least expected use of veterinary care, which is of great importance to correctly characterize the distribution of cancer cases (20).

The precise determination of the CA is important for the statistical soundness of posterior inferences. This is beneficial for the correct identification of the population at risk and prevents biases in subsequent analysis. This is true, for example, in the spatial analysis which is sensitive to the size and shape of the area of analysis (53) and incomplete case reporting (54). Misspecification of the underlying model may also have a significant impact on the CA estimation, which is why we tested different likelihood distributions in order to find the best performance on a spatially autoregressive conditional zero-inflated negative binomial model. The parameterization of this model takes into account the spatial correlation between CT and differentiate between zeros representing under-ascertainment and lack of cases (55), which have been considered relevant issues when assessing cancer incidence (10, 12). The better performance of the CAR model suggests the existence of spatial effects in the data and is consistent with the effects later associated by the logistic regression with the inclusion in the CA (distance and income), as they are likely to have a spatial component. CTs with similar distances to the hospital may be correlated or high-/low-income households may be clustered in specific groups of CT. Although we are not aware of other data-driven CA estimations for cat and dog cancer, the benefits of models considering spatial components over conventional approaches have also been shown when studying dog cancer incidences, having a greater influence on a local scale (11). This indicates the need to contemplate spatial clustering when dealing with this type of data.

While the methodology applied in this study worked reasonably well, there are also some limitations to this approach. The most important of which is the lack of reliable demographic data, which is a persistent problem in cancer studies in companion animals (7). Since no census was available, we calculated the population data from the estimated average number of dogs and cats by household in the state of California (35). Therefore, we expect some degree of bias, but overall, we believe this can give a realistic approximation. However, this issue is more problematic if one intends to include additional demographic data. For example, factors such as sex, age, or breed are well-known risk factors for the incidence of cancer (56, 57) and could be very useful to obtain a better characterization, but could not be used here due to the lack of population information. Therefore, this highlights the need for better sources of population data in veterinary epidemiology. The development of veterinary registries of disease events and population demographics would constitute a valuable tool that would help veterinary practitioners in the diagnosis and control of diseases by providing information controlled for important characteristics such as breeds, gender, or age groups and related to the geographic area (7). Our work may contribute to helping the identification of factors affecting data collection and so in data standardization, but major challenges are still ahead such as the frequent lack of extensive census or the lack of a unique single coding system, for example for cancer disease different from humans (7), that are necessary to address to improve the quality of the available data.

We found that distance to the hospital was the main contributor to the estimated CA, which is expected and has traditionally been the main parameter considered when establishing a CA. However, our results highlighted the contribution of additional factors since adding the average income with an interaction term significantly improved the prediction of the CA. Other factors such as the distance to other hospitals, their density, or the presence of geographical barriers may affect a hospital CA (10, 12, 58) and their inclusion may have improved these estimations. Using these data-driven CAs allows the identification of those factors that influence the arrival of cases in specific hospitals, which is valuable since the influential factors and the magnitude of their effect may vary significantly between hospitals (21, 22). The methodology described in this study may be helpful to achieve a better characterization of factors that, by influencing the presentation of cases, may cause biases and confounding effects in the analysis of data collected by hospitals. Thus, it may improve the accuracy and reliability of posterior inferences.

Bayesian hierarchical models present specific advantages to analyzing this type of dataset since they allow control of the influential covariates, include previous knowledge, specifying different spatial relationships among the observations, etc. However, despite the increased use of these approaches in spatial epidemiology in the last decades in the context of medical research and public health (32), the application of Bayesian frameworks to CA estimation has only been carried out in human medicine where its use is still very limited (25, 59). Bayesian methods have the potential to characterize a CA. However, they can be limited by the scarce availability of population data for dogs and cats. This may prevent the good identification of the model, its predictability, and the extrapolation of models to other areas where less population information is available. It is also noteworthy that the application of this methodology is not limited to hospital registries but may be useful for other data registries. For example, laboratory results, which have also been an important source of disease data, also face similar bias submission problems.

In conclusion, the usefulness of registry data depends on the quality of the characterization of the population at risk (7, 49) and a starting point of a good analysis is the correct understanding of the origin of the data and the factors that influenced their collection. For this purpose, the available literature lacks a dominant method to estimate CA but, in this study, we have shown the application of a data-driven Bayesian framework to delineate catchments in veterinary hospitals. This methodology has worked well to characterize the catchment of cancer cases and visits, and it can be easily adjusted to other diseases, centers, and needs. Considering our results, the application of this type of method could be of great interest to explore and rule out possible biases in the collection of data and improve the accuracy of analysis and inferences based on registry data.

## Data Availability Statement

The raw data supporting the conclusions of this article will be made available by the authors, without undue reservation.

## Author Contributions

Conceptualization and supervision: BM-L. Data acquisition: MK. Data curation: RR and JD. Statistical analysis and writing the original draft: JD. Critical review of work: BM-L and MK. All authors read and approved the final manuscript.

## Funding

This work was supported in part by funds from the UC Davis NCI Designated Comprehensive Cancer Center under the project Building a Veterinary Cancer Registry (P30CA093373) and a postdoctoral grant to JD (Axudas de apoio á etapa de formación posdoutoral, ref. 2019-HG005, Xunta de Galicia, Spain).

## Conflict of Interest

The authors declare that the research was conducted in the absence of any commercial or financial relationships that could be construed as a potential conflict of interest.

## Publisher's Note

All claims expressed in this article are solely those of the authors and do not necessarily represent those of their affiliated organizations, or those of the publisher, the editors and the reviewers. Any product that may be evaluated in this article, or claim that may be made by its manufacturer, is not guaranteed or endorsed by the publisher.
