# Supplementary material for: Application of Bayesian Regression for the Identification of a Catchment Area for Cancer Cases in Dogs and Cats
Source: Front Vet Sci. 2022 Jul 25;9:937904. doi: 10.3389/fvets.2022.937904 (PMC9359078; doi:10.3389/fvets.2022.937904)
Supplement: Supplementary file 1 [file Data_Sheet_1.PDF]

Code for analysis

PREVIOUS PROCESSING

For conditional autoregressive models a matrix of locations is needed. This is not necessary in models that do not use a CAR component. This requires a map. To handle it, we are using here a Spatial Polygon Data Frame created with the package sp

```
load(map)
head(map) # example to see the structure
# We will identify our polygons with the variable "CensusTract"
class       : SpatialPolygonsDataFrame
features    : 6
extent      : -122.0173, -121.9193, 37.49557, 37.54419  (xmin, xmax, ymin, ymax)
crs         : +proj=longlat +ellps=GRS80 +towgs84=0,0,0,0,0,0,0 +no_defs
variables   : 25
names       : CensusTract, COUNTYFP, STATEFP,  ...
min values  : 06001442700,      001,      06,  ...
max values  : 06001443102,      001,      06,  ...

library(spdep) # A collection of functions to create spatial weights matrix objects from
# polygon 'contiguities'
nb<-poly2nb(map,row.names = map@data$CensusTract) # creates a neighbour list. We give them
# the names in CensusTract

#example
head(nb)
[[1]] # polygons that are neighbors with polygon 1 and so on ...
[1]    2 1860 1890 1916 1953 2626 2628 2646
[[2]]
[1]    1    3  572 1860 1915 1916 2626 2628
M<-nb2mat(nb,style="B")# generates a weights matrix for a neighbours list with spatial
# weights for the chosen coding scheme ("B" in this case)
# The rows are the CensusTract label and matrix to indicate whether a pair is neighbor or
# not
```

BAYESIAN REGRESSION USING STAN AND BRMS

```
model<-brm(visits ~ species + period + #fixed factors
             offset(log(pop)) + #adding an offset to obtain RR
             (1 | Census tract) +#random effect
             ## the next car function is only needed if you intend to run a conditional
# autoregressive model. Do not use this function in the model otherwise
             car(M, # matrix of locations
                 gr = Census tract, #grouping factor
                 type = "bym2"), #type of CAR structure. You'll find more types of
```

```
# structures with ?car()
           data=dataBR, #data for the regression
           data2=list(M=M),# data for the car function, obtained in the previous step. Not
# needed if no CAR regression is done
           family=zero_inflated_negbinomial,# specify the type of regression. You'll find
# different types and details with ?brmsfamily
           chains=4, # specify the number of Markov chains.
           iter=2000, # specify the number of iterations per chain, including warmup
           warmup=floor(iter/2))# specify warmup (burnin)
```

DIAGNOSTICS

We use shinystan to assess the model performance

```
library(shinystan)
launch_shinystan(model)# offers a graphic interface in which Bayesian diagnostics can be
# observed
```

COMPARE MODELS

```
libray(loo)
loo_model<-loo(model,save_psis=TRUE)
loo_model_n<-loo(model,save_psis=T) #alternative models
loo_compare(loo_model,loo_model_n)
# more details in Vehtari et al., 2017 doi: 10.1007/s11222-016-9696-4
```

GET THE CATCHMENT AREA

```
get_CA<-function(model){
  #aggregation: CensusTract
  sims <- as.matrix(model) #get all the values for each regression parameter
  u_sims <- sims[, 2717:5423] # get the columns of interest (it depends on the model)
  exp_u_sims <- exp(u_sims) # exponentiate them
  prob.nb <- apply(exp_u_sims, 2, function(x) sum(x > 1))
  prob.nb2 <- data.frame(prob.nb/nrow(sims))
  #t<-aggregation
  a <- levels(as.factor(model$data$CensusTract))#change accordingly
  prob.nb3 <- cbind(prob.nb2, a)
  prob.nb3$filter <- with(prob.nb3, ifelse(prob.nb.nrow.sims. < 0.9, 0, 1))
  colnames(prob.nb3)[2] <- "CensusTract" #name in the map
  prob.nb3
}
```
